# Associations between Changes in City and Address Specific Temperature and QT Interval - The VA Normative Aging Study

**DOI:** 10.1371/journal.pone.0106258

**Published:** 2014-09-19

**Authors:** Amar J. Mehta, Itai Kloog, Antonella Zanobetti, Brent A. Coull, David Sparrow, Pantel Vokonas, Joel Schwartz

**Affiliations:** 1 Exposure, Epidemiology and Risk Program, Department of Environmental Health, Harvard School of Public Health, Boston, Massachusetts, United States of America; 2 The Department of Geography and Environmental Development, Ben-Gurion University of the Negev, Beer Sheva, Israel; 3 Department of Biostatistics, Harvard School of Public Health, Boston, Massachusetts, United States of America; 4 The VA Normative Aging Study, Veterans Affairs Boston Healthcare System, Boston, Massachusetts, United States of America; 5 The Channing Laboratory, Brigham and Women's Hospital, Harvard Medical School, Boston, Massachusetts, United States of America; 6 Department of Medicine, Boston University School of Medicine, Boston, Massachusetts, United States of America; University of Adelaide, Australia

## Abstract

**Background:**

The underlying mechanisms of the association between ambient temperature and cardiovascular morbidity and mortality are not well understood, particularly for daily temperature variability. We evaluated if daily mean temperature and standard deviation of temperature was associated with heart rate-corrected QT interval (QTc) duration, a marker of ventricular repolarization in a prospective cohort of older men.

**Methods:**

This longitudinal analysis included 487 older men participating in the VA Normative Aging Study with up to three visits between 2000–2008 (n = 743). We analyzed associations between QTc and moving averages (1–7, 14, 21, and 28 days) of the 24-hour mean and standard deviation of temperature as measured from a local weather monitor, and the 24-hour mean temperature estimated from a spatiotemporal prediction model, in time-varying linear mixed-effect regression. Effect modification by season, diabetes, coronary heart disease, obesity, and age was also evaluated.

**Results:**

Higher mean temperature as measured from the local monitor, and estimated from the prediction model, was associated with longer QTc at moving averages of 21 and 28 days. Increased 24-hr standard deviation of temperature was associated with longer QTc at moving averages from 4 and up to 28 days; a 1.9°C interquartile range increase in 4-day moving average standard deviation of temperature was associated with a 2.8 msec (95%CI: 0.4, 5.2) longer QTc. Associations between 24-hr standard deviation of temperature and QTc were stronger in colder months, and in participants with diabetes and coronary heart disease.

**Conclusion/Significance:**

In this sample of older men, elevated mean temperature was associated with longer QTc, and increased variability of temperature was associated with longer QTc, particularly during colder months and among individuals with diabetes and coronary heart disease. These findings may offer insight of an important underlying mechanism of temperature-related cardiovascular morbidity and mortality in an older population.

## Introduction

It is well established that increases and decreases in ambient mean temperatures are associated with mortality [1], [2], and that elderly age, chronic disease including cardiovascular disease and diabetes mellitus, mental illness, sociodemographic characteristics, and social isolation may heighten susceptibility to temperature-related mortality [3]–[6]. While the highest risk of heat-related mortality is for mean temperature measured within 24-hours and up to 3 days before event [1], [3], [7], cold effects on mortality continue longer. In general there is limited evidence demonstrating risk of mortality associated with changes in daily mean temperature averaged over longer intervals (e.g. between 7 and 40 days) [1], [8]. Recent studies also demonstrate that variability of and large changes in the daily mean temperature averaged over shorter and longer intervals are also associated with mortality [9]–[12]. Finally, most epidemiologic studies to date have used a single value for a city to characterize exposure, which ignore important differences that may influence temperature at individual residences including urban heat islands, distance from water, and amount of impermeable surface [13].

There are clear physiological mechanisms for the association of extreme heat or cold with mortality, particularly the reduced ability to regulate core temperature [3]. However, the underlying biological mechanisms behind the associations observed between mean temperature and mortality under more moderate conditions, and over longer intervals, are not well understood, and may be distinct from those associated with mean temperature and mortality under more severe conditions and shorter intervals. Understanding these mechanisms, especially in vulnerable populations, may lead to more focused intervention measures and improved risk assessment. Previous research has demonstrated associations between mean temperature, averaged over shorter and longer intervals, and markers of inflammation [14]–[16], hemodynamics [17]–[18], and cardiac autonomic function [19]–[23], which suggests that these pathways may be potential mechanisms of mean temperature-related cardiovascular morbidity and mortality. It also hypothesized by Hampel and colleagues [24] that ambient mean temperature may affect repolarization, including QT interval and T-wave abnormalities, which are associated with arrhythmic events and cardiovascular mortality [25]–[28]. A previous study conducted within a subset of myocardial infarction survivors of the KORA cohort in Augsburg, Germany observed a U-shape association between ambient mean temperature and T-wave amplitude, at all lags between 0 and 5 days, with the highest T-wave amplitude at 5°C [24]. In the same study, investigators did not identify any association between mean temperature and QTc. Whether changes in mean temperature averaged over longer time periods affects QTc is not known. Additionally, very little is known of the underlying mechanisms that may explain the association between morbidity and mortality associated with temperature variability. It is hypothesized that increased temperature variability may stress the ability of the thermoregulation system to adapt to sudden and extreme temperature changes, and that adaptive ability is reduced in individuals who are immunocompromised or present with chronic illness [29].

For this study, we evaluated if changes in the daily mean temperature, and the daily standard deviation of temperature, were associated with heart-rate corrected QT interval (QTc) in a cohort of elderly males in the Boston Metropolitan Area. Additionally, we applied a validated spatially and temporally resolved prediction model utilizing satellite surface temperature data [13] to estimate daily air temperature at the participant's home address, and compared associations for daily mean temperature measured from a local weather monitor and estimated from the predicted model.

## Materials and Methods

### Ethics Statement

The present study was approved by the Human Research Committees of the Harvard School of Public Health and the Department of Veterans Affairs Boston Healthcare System, and written informed consent was obtained from participants prior to participation.

### Study population and design

Participants included in this analysis were enrolled in the Veteran Affairs Normative Aging Study (NAS), an ongoing longitudinal study of aging established in 1963, details of which have been published previously [30]. Briefly, the NAS is a closed cohort of 2,280 male volunteers from the Greater Boston area aged 21–80 years at entry, who were enrolled after an initial health screening determined that they were free of known chronic medical conditions. The men have been reevaluated every 3 to 5 years by using detailed on-site physical examinations and questionnaires. Dropout has been less than 1% per year in this cohort and predominantly occurs when participants move out of the study area. The other major reason for loss to follow-up has been mortality.

From November 2000 to December 2008, there were 694 participants who underwent electrocardiogram (ECG) monitoring, from whom 580 individuals have valid QT measurements at one or more visits to give a total of 926 QT measurements. We excluded 14 participants with incomplete information on relevant covariates, 42 participants with incomplete address history for estimating temperature from the spatiotemporal model, and 37 participants with pacemakers or ECGs indicating atrial fibrillation, leaving a total of 743 measurements from 487 participants. For the purpose of this analysis, the term ‘baseline visit’ refers to the first visit when QT was measured.

### ECG measurement and analysis

The ECG was recorded at the exam site (VA Boston Healthcare System, Boston, MA) for 5 to 10 min between 0530 and 1400 hours with a two-channel (five lead) ECG monitor (Trillium 3000; Forest Medical, Inc., East Syracuse, NY) using a sampling rate of 256 Hz per channel. A detailed description of the protocol is provided elsewhere [31], [32]. The electrocardiogram digital recordings were processed using personal computer-based software (Trillium Platinum Holter Analysis Software for MS Windows; Forest Medical) to create a Mathcad (Parametric Technology Corporation, Needham, Massachusetts) file containing QT interval measurements. A Win32 console application (Microsoft Corporation, Redmond, Washington) was used to obtain QT and QTc values from the data. This application measured the QT interval from the beat onset to the end of the T wave only on normal or supraventricular beats and calculated the QTc value in milliseconds (msec) using Bazett's formula as described by Bednar et al. [33]. The QT interval was not calculated if the T wave did not have sufficient amplitude, as determined by the program algorithm. The Intraclass correlation coefficient for mean QTc across all visits was 0.65, indicating moderate agreement.

### Meterology, pollution, and spatiotemporal assessment of temperature

Hourly temperature and humidity data were obtained from a stationary monitor located at the Boston Logan airport weather station (12 km from the exam site), and we calculated the 24-hour mean and standard deviation of temperature for each calendar day in the study period. For this analysis, we evaluated moving average windows of 24-hr mean temperature and 24-hr standard deviation of temperature of 1 and up to 7 days prior to visit, as well as 14, 21, and 28 days prior to visit.

Ambient particulate pollutant concentrations were monitored at our Harvard Air Pollution Supersite located near downtown Boston 1 km from the exam site, and for the present analysis we considered evaluation of 4-hour lag black carbon (BC) exposure prior to visit, which was identified as a significant predictor of QTc in a previous analysis [34]. Hourly BC concentrations were measured using an Aethalometer (Magee Scientific Company, Model AE-16, Berkeley, CA). BC is associated with traffic emissions especially those related to diesel fuel combustion.

Predicted air temperature exposure data were generated by using a novel air temperature spatiotemporal prediction model [13]. Our air temperature predictions are generated by a series of statistical models. In brief, we start by using a mixed model approach to calibrate daily MODIS surface temperature in each grid cell where both air temperature and surface temperature values within 1 km are available on that day. This model is fit by regressing monitored air temperature against surface temperature with additional air temperature predictors (percent urban, elevation, normalized difference vegetation index) as well as a random intercept and a random slope for surface temperature for each day. Since surface temperature values are often missing due to cloud cover or retrieval errors, the base model often fails to provide predictions for many grid cell-day combinations. To estimate air temperature when no surface temperature data are available, we fit an additional model that takes advantage of the association of grid cells surface temperature values with air temperature monitoring located elsewhere and the association with surface temperature values in neighboring grid cells. Importantly, since daily air temperature varies considerably between different geographical regions, we built a 60 km buffer around each monitor and used its daily temperature as a predictor of temperature in all cells that fell within the buffer. Out-of-sample “ten-fold” cross-validation was used to quantify the accuracy of our predictions. Our model performance was excellent for both days with available satellite data and days without satellite observations (mean out-of-sample R^2^ = 0.947 and R^2^ = 0.940, respectively). To estimate air temperature exposure at each participant's residence, we linked each participant's residence to the corresponding model 1×1 km grid cell in which it fell. When we regressed monitored temperature as a function of predicted temperature for the monitors within 150 km of Boston that were left out from the analysis, the calculated root mean square predicted error (RMSPE) was 1.48. In contrast when we calculated the RMSPE by using the Logan weather station (i.e. local monitor) readings, the RMSPE was 2.3, almost a full degree difference. This contrast demonstrates the improved accuracy of spatially resolved temperature estimates from our models for NAS participants living further from central Boston. Similar to 24-hr mean temperature measured from the local monitor, we evaluated moving average windows of the 24-hr mean temperature estimated from the prediction model at 1 and up to 7 days prior to visit, as well as 14, 21, and 28 days prior to visit.

### Statistical analysis

All statistical analyses were carried out using SAS Version 9.2 (SAS Institute, Cary, NC) and R (R Foundation for Statistical Computing, Vienna, Austria). We used linear mixed-effects regression (PROC MIXED) with random participant-specific intercepts, accounting for the correlation of repeated QTc measures across time points [35], to model mean QTc as a continuous function of moving average of 24-hr mean temperature measured from the local monitor, 24-hr mean temperature estimated from the predictive model, and 24-hr standard deviation of temperature. Estimates are given per interquartile range of the temperature variable for the specific moving average. In separate models, we fit each moving average window of each temperature variable one at a time in an established confounder model consisting of fixed characteristics including age at baseline visit, race (black, white as reference), and years of education, and of time-varying characteristics that were ascertained at each visit including years since baseline visit, body mass index (bmi) (kg/m^2^), total cholesterol (mg/dL), mean arterial pressure (mmHg), diabetes status (physician's diagnosed diabetes or fasting blood glucose>126 mg/dL [yes, no as reference]), QT prolonging medication including beta-blockers (yes, no as reference), percent of census tract ≥25 years of age without high school diploma, percent of census tract that is non-white, alcohol consumption (≥2 drinks/day, <2 drinks/day as reference), smoking status (current, former, never as reference), day of week, seasonality (using sine and cosine), 24-hour mean relative humidity, and 4-hour lag BC concentration (µg/m^3^). To identify QT-prolonging medications, we considered drugs grouped by risk of torsades and possible risk of torsades from the Arizona Center for Education and Research on Therapeutics [36]. The above covariates were chosen a priori as potential confounders or important determinants of QTc. We investigated possible effect modification by season (warm: April–September; cold: October-March), obesity (bmi≥30 kg/m^2^), age, diabetes, and coronary heart disease, by adding an interaction term for the temperature and modifier variables to the models. Two-sided p-values≤0.05 were interpreted as statistically significant for main and interaction terms.

In a preliminary analysis, we evaluated whether the association between the moving average of each temperature variable and QTc was non-linear. We fit generalized additive mixed models with penalized splines for each of the temperature variables. The generalized cross validation criterion of the model was used to determine the degrees of freedom of the spline for the nonlinear term, and we compared the Akaike information criterion to assess model fit between models with the linear and non-linear term. For all models, the linear term for each of the temperature variables was the better fit. Similarly, we fit penalized splines for years since baseline visit, age at baseline visit, body mass index, mean arterial pressure and total cholesterol one at a time in generalized additive mixed models to evaluate whether the association between these covariates and QTc were non-linear. The linear term for each of these covariates was the better fit.

### Secondary analysis

Of the 487 participants included in this analysis, 290 (59.7%) did not participate in a subsequent follow-up visit. Because healthier men may be more likely to participate in subsequent follow-up visits, we applied inverse probability weights to correct for this potential survival bias as a sensitivity analysis [37]. Similar to methods described by Zanobetti et al. [38], we used logistic regression to calculate the probability of having a second and third subsequent follow-up visit given the following factors at the previous visit: age, body mass index, smoking status and pack-years, hypertension, cholesterol and diabetes. We then used the inverse of these predicted probabilities as the weights for those person-visits in the linear mixed-effect models. The inverse probability weight for all participants at the baseline visit was ‘1’.

## Results

The 487 participants included in this analysis had a total of one (n = 290), two (n = 138), or three (n = 59) separate visits with valid QTc measurements (Table 1). The mean age at baseline visit when QTc was measured was approximately 74 years. The mean QTc measured at the baseline visit approximated 392 msec. The distributions of QTc measured in all visits during warm (Mean (SD): 390.7 msec (24.6)) and cold (Mean (SD): 391.4 msec (26.0)) months closely approximated each other. Participants were predominantly white, former smokers, and hypertensive.

**Table 1:** Characteristics of study participants at first visit, the VA Normative Aging Study, November 2000 to December 2008.

Characteristics	n (%)
Physician diagnosed Diabetes Mellitus or fasting blood glucose>126 mg/dL	92 (18.9)
Coronary heart disease	133 (27.3)
Hypertension	342 (70.2)
Use of QT prolonging medication	180 (36.9)
Obesity	
BMI≥30 kg/m^2^	118 (24.2)
BMI<30 kg/m^2^	369 (75.8)
Race	
Black	13 (2.7)
White	474 (97.3)
Alcohol consumption (drinks/day)	
≥2	93 (19.1)
<2	393 (80.9)
Smoking status	
Former	329 (67.6)
Current	23 (4.7)
Never	135 (27.7)
Number of visits with QTc measured	75 (20.3)
One	290 (59.7)
Two	138 (28.3)
Three	59 (12.1)
	Mean (SD)
QTc, msec	391.7 (26.2)
Age, years	73.6 (6.7)
Body mass index, kg/m^2^	27.9 (4.0)
Total cholesterol, mg/dL	191.2 (36.9)
Mean arterial pressure, mmHg	92.4 (11.0)
Maximum years of education	15.0 (3.0)

Table 2 summarizes the distribution of the meteorology and pollution parameters and correlations between them in all person-visits included in this analysis. 24-hr mean temperature as measured from the local monitor and as estimated from the spatiotemporal predictive model closely approximated each other, and as expected, a very strong positive correlation was also observed. Weaker correlations were observed between both 24-hour mean temperature variables with 24-hour mean relative humidity, 4-hour lag BC, and 24-hr standard deviation of temperature.

**Table 2:** Summary statistics and Pearson Correlation Coefficients of meteorologic and black carbon measurements at all visits (n = 743), the VA Normative Aging Study, November 2000 to December 2008.

	Summary Statistics			*r*			
	Mean (SD)	Median (IQR)	24-hr T_S_	24-hr RH	24-hr TSD_S_	24-hr T_P_	4-hr lag BC
24-hr T_L_, °C	11.5 (9.0)	11.6 (4.0, 18.8)	1.00	0.12	0.24	0.94	0.19
24-hr RH,%	66.5 (15.2)	66.5 (54.3, 77.9)		1.00	−0.34	0.20	0.12
24-hr TSD_L_, °C	2.4 (1.2)	2.3 (1.5, 3.0)			1.00	0.11	−0.05
24-hr T_P_, °C	11.4 (5.4)	11.5 (7.3, 15.8)				1.00	0.23
4-hr lag BC, µg/m^3^	0.7 (0.5)	0.5 (0.3, 0.9)					1.00

Abbreviations: SD – standard deviation; IQR – interquartile range; 24-hr T_L_ – 24-hour mean temperature measured from local weather monitor; 24-hr RH – 24-hour mean relative humidity measured from local weather monitor; 24-hr TSD_L_ – 24-hr standard deviation of temperature measured from local weather monitor; 24-hr T_P_ – 24-hour mean temperature measured from prediction model; BC – 4-hour lag black carbon measured from central monitoring site.

**Figure 1:**
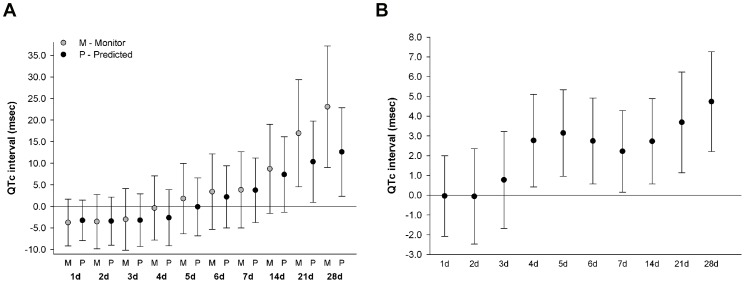
Associations between QTc and 1 interquartile range increase of moving average 24-hr mean temperature measured from local monitor or estimated from predictive model (1a) and 24-hr standard deviation of temperature measured from local monitor (1b). Associations were estimated in linear mixed-effect regression models with random intercept for study participant and adjusted for years since baseline visit, age at baseline visit, race, body mass index, total cholesterol, mean arterial pressure, diabetes, QT prolonging medication, years of education, percent of census tract ≥25 years of age without high school diploma, percent of census tract that is non-white, alcohol consumption, smoking status, day of week, seasonality, 24-hour mean relative humidity, and 4-hour lag black carbon concentration.

Higher 24-hr mean temperature, as measured from the local monitor, was significantly associated with longer QTc at moving averages of 21 and 28 days (Figure 1a). Specifically, interquartile range (IQR) increases in 21-day (15.1°C) and 28-day (15.2°C) moving average of 24-hr mean temperature measured from the local monitor was associated with a 17.0 msec (95%CI: 4.5–29.4) and 23.1 msec (95%CI: 9.0–37.2) longer QTc, respectively. While significant associations were present at the same moving averages for 24-hr mean temperature estimated from the spatiotemporal predictive model, the effect sizes were moderately smaller in comparison (Figure 1a); 8.4°C and 8.5°C IQR increases in 21 and 28-day moving average of 24-hr mean temperature were associated with a 10.4 msec (95%CI: 0.9, 19.8) and 12.6 msec (95%CI: 2.4, 22.9) longer QTc, respectively. Across all moving averages, the standard errors for the associations between 24-hr mean temperature and QTc were smaller when utilizing the spatiotemporal predictive model rather than measurements from the local monitor. While marginally statistically significant associations (p<0.10) were observed between higher 14-day moving average of 24-hr mean temperature and longer QTc utilizing both approaches for estimating mean temperature, no associations were observed for 24-hr mean temperature at shorter moving average windows between 1 and 7 days.

Statistically significant associations were also observed between 24-hr standard deviation of temperature and QTc from moving averages of 4 days up to 28 days (Figure 1b). A 1.9°C IQR increase in the 4-day moving average 24-hr standard deviation of temperature was associated with a 2.8 msec (95%CI: 0.4, 5.2) longer QTc. The strongest associations between higher 24-hr standard deviation of temperature and longer QTc was observed at the 28-day moving average; a 0.5°C IQR increase in the 28-day moving average of 24-hr standard deviation of temperature was associated with a 4.7 msec (95%CI: 2.2, 7.3) longer QTc.

As part of a sensitivity analysis, no considerable changes in the associations between 24-hr mean temperature and 24-hr standard deviation of temperature and QTc, as shown in Figures 1a and 1b, were observed after inverse probability weights were applied (data not shown). Additionally, there were no appreciable changes in the effect estimates or standard errors for moving average 24-hr mean temperature in minimally adjusted linear mixed-effect models that retained a reduced set of covariates including age at baseline visit, seasonality, 24-hr mean relative humidity, and 4-hr lag black carbon concentration in the models (Table S1). However, marginally stronger positive associations with wider standard errors were observed for 24-hr standard deviation of temperature at moving averages of 14, 21, and 28 days, in the minimally adjusted model in comparison with the effect estimates shown in Figure 1b (Table S1).

**Figure 2:**
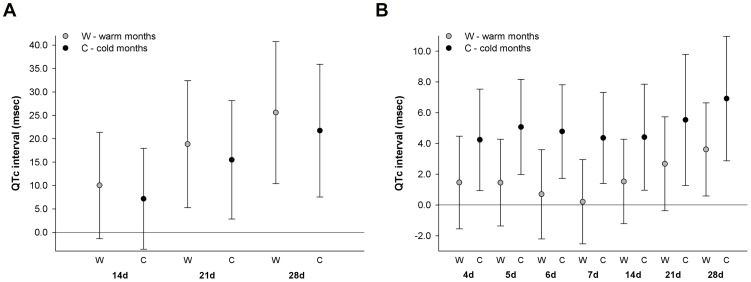
Associations between QTc and 1 interquartile range increase of moving average 24-hr mean temperature (2a) and 24-hr standard deviation of temperature (2b) measured from local monitor by season. Associations were estimated in linear mixed-effect regression models with random intercept for study participant and adjusted for years since baseline visit, age at baseline visit, race, body mass index, total cholesterol, mean arterial pressure, diabetes, QT prolonging medication, years of education, percent of census tract ≥25 years of age without high school diploma, percent of census tract that is non-white, alcohol consumption, smoking status, day of week, seasonality, 24-hour mean relative humidity, and 4-hour lag black carbon concentration; associations between moving average temperature variable and QTc for warmer months and colder months were estimated from interaction models.

At moving averages of 14, 21, and 28 days, the associations between 24-hr mean temperature, as measured from the local monitor, and QTc as shown in Figure 1a were slightly stronger during warm than cold months (Figure 2a). However, the confidence intervals for the estimated associations for warm and cold months widely overlapped, and effect modification by season was not statistically significant. A similar pattern in comparing estimated associations between warm and cold months was observed for 24-hr mean temperature predicted from the spatiotemporal model (Figures S1a in File S1). In contrast, stronger associations were observed between 24-hr standard deviation of temperature and QTc in cold than warm months (Figure 2b). The statistically significant associations between elevated 24-hr standard deviation of temperature and longer QTc at moving average windows between 4 and 7 days, as shown in Figure 1b, were observed only in cold months. Effect modification by season on the association between 24-hr standard deviation of temperature and QTc was statistically significant at moving averages of 6 (p_interaction_ = 0.05) and 7 days (p_interaction_ = 0.04).

**Figure 3:**
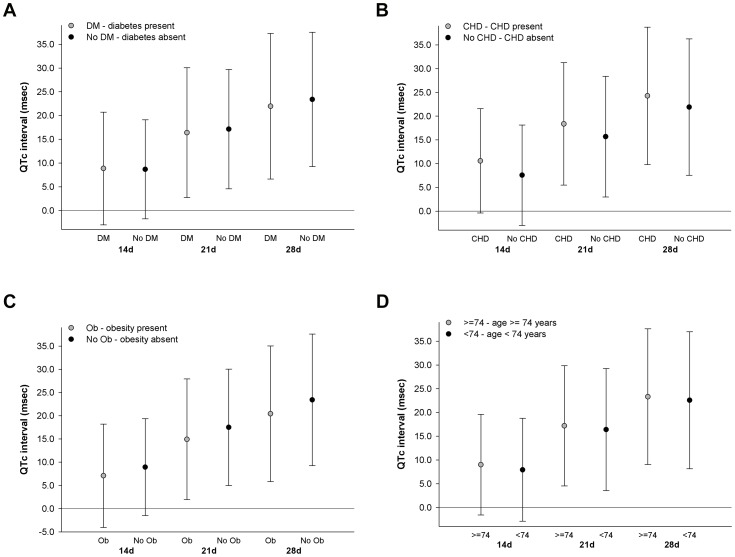
Associations between QTc and 1 interquartile range increase of moving average 24-hr mean temperature measured from local monitor estimated in subgroups defined by diabetes (3a), coronary heart disease (3b), obesity (3c), and age (3d). Associations were estimated in linear mixed-effect regression models with random intercept for study participant and adjusted for years since baseline visit, age at baseline visit, race, body mass index, total cholesterol, mean arterial pressure, diabetes, QT prolonging medication, years of education, percent of census tract ≥25 years of age without high school diploma, percent of census tract that is non-white, alcohol consumption, smoking status, day of week, seasonality, 24-hour mean relative humidity, and 4-hour lag black carbon concentration; associations between moving average 24-hr mean temperature and QTc in each subgroup were estimated from interaction models.

**Figure 4:**
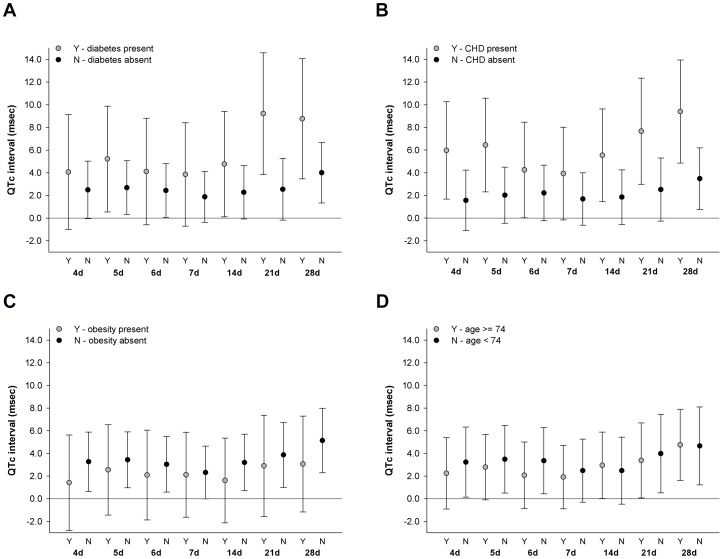
Associations between QTc and 1 interquartile range increase of moving average 24-hr standard deviation of temperature measured from local monitor estimated in subgroups defined by diabetes (3a), coronary heart disease (3b), obesity (3c), and age (3d). Associations were estimated in linear mixed-effect regression models with random intercept for study participant and adjusted for years since baseline visit, age at baseline visit, race, body mass index, total cholesterol, mean arterial pressure, diabetes, QT prolonging medication, years of education, percent of census tract ≥25 years of age without high school diploma, percent of census tract that is non-white, alcohol consumption, smoking status, day of week, seasonality, 24-hour mean relative humidity, and 4-hour lag black carbon concentration; associations between moving average 24-hr standard deviation of temperature and QTc in each subgroup were estimated from interaction models.

Estimated associations between 24-hr mean temperature and 24-hr standard deviation of temperature, as measured from the local monitor, and QTc by subgroup according to individual-level characteristics, including age ≥74 years, obesity, coronary heart disease, and diabetes, are presented in Figures 3 and 4. Significant effect modification by age ≥74 years, obesity, coronary heart disease, and diabetes, on the associations between 24-hr mean temperature and QTc at moving averages of 14, 21, and 28 days, was not observed. A similar pattern in comparing estimated associations between subgroup categories was observed for 24-hr mean temperature predicted from the spatiotemporal model (S1b-S1e in File S1). Across moving averages of 4 and up to 28 days, the positive associations between higher 24-hr standard deviation of temperature and longer QTc were more pronounced among participants with diabetes and coronary heart disease, and there was significant effect modification observed by diabetes at a moving average of 21 days (p_interaction_ = 0.02, Figure 4a) and by coronary heart disease at moving averages of 21 (p_interaction_ = 0.05, Figure 4b) and 28 days (p_interaction_ = 0.02, Figure 4b).

## Discussion

In this sample of predominantly older white men, we observed that higher daily mean temperature was associated with longer QTc, specifically at longer moving average windows of 21 and 28 days while no associations were observed at shorter moving averages. In contrast, increased daily standard deviation of temperature was associated with longer QTc at both shorter and longer averaging moving averages, spanning from 4 and up to 28 days, particularly in the colder months for shorter moving averages and in individuals with diabetes and coronary heart disease at longer moving averages.

Consistent with the previous study in the subset of myocardial infarction survivors of the KORA cohort [24], we did not observe any association between mean temperature and QT interval at moving averages of 1 to 7 days. Previous studies to date have not examined daily temperature variability in association with QTc. The associations between higher 24-hr standard deviation of temperature and longer QTc at moving averages of 4 to 7 days, as shown in Figure 1b, were similar in magnitude even after adjustment for corresponding moving average of 24-hr mean temperature (data not shown), suggesting that the variability of temperature, rather than the mean temperature, averaged over shorter time periods may be more relevant for changes in QTc. An alternative explanation for absence of association between QTc and 24-hr mean temperature averaged over a shorter time period is that there may be less exposure measurement error of mean temperature with longer averaging times than with shorter averaging times.

Non-linear associations including J-, V-, or U-shaped associations between temperature and mortality have also been observed previously [1], [39], [40], and in our preliminary analysis of the present study we did not observe non-linear associations between temperature and QTc. Additionally, a previous analysis in the VA Normative Aging Study did not identify any evidence of a non-linear association between apparent temperature and heart rate variability [21]. Similarly, the previous study conducted within the KORA cohort did not find evidence for a deviation of linearity of the association between temperature and QTc [24].

The underlying biological mechanism(s) that may explain the association between temperature and QTc is not known. A previous analysis of the Normative Aging Study showed that 14-day moving averages of daily mean apparent temperature were associated with reduced heart rate variability, a marker of cardiac autonomic dysfunction, during warm months [21]. There is also observational evidence to support the association between reduced heart rate variability and autonomic dysfunction with prolonged QT interval [41]–[44]. Taking these findings together, we hypothesize that the association between mean temperature and QTc may be mediated by autonomic dysfunction.

A main advantage of the present study was the ability to compare associations between moving average of 24-hr mean temperature and QTc utilizing the local monitor and the spatiotemporal predictive model using satellite data. However, very little difference was observed between the two methods at the shorter moving averages, suggesting that geographical differences do not explain much of the variation in QTc in response to daily mean temperature. While more divergence in the estimated associations between the two methods was observed at the longer moving average windows, this may be an artifact of larger variation in mean temperature measured from the fixed monitor compared with the spatiotemporal predictive model. Additionally, the standard errors of the estimated associations for moving average of 24-hr mean temperature were reduced when utilizing the spatiotemporal predictive model, suggesting that utilizing measurements of daily mean temperature from a single site may result in Berkson measurement error, which has been observed more commonly with air pollution [45], rather than classical exposure measurement error.

This study has a number of strengths including prospective design, spatiotemporal assessment of mean temperature, methods to address selection bias, and adjustment for multiple confounders, but there are several limitations that should be considered. The 24-hour mean temperature as predicted from the model is based on daily MODIS surface temperature rather than hourly estimates of surface temperature. To predict the 24-hour standard deviation of temperature based on hourly estimates of surface temperatures would be computationally intensive and not feasible for this study. Assuming the estimation for daily standard deviation of temperature from a spatiotemporal predictive model is more valid than measured from a local monitor, we hypothesize that the observed associations between moving averages of 24-hr standard deviation of temperature and QTc would have reduced standard errors, as observed for the associations between moving averages of 24-hr mean temperature and QTc. Our study also used a predefined computer algorithm that automatically detected beat labels and QT intervals, rather than use manual detection, to analyze the ECG recordings. Thus, the outcome assessment of QTc is less susceptible to differential measurement errors and inter-technician variability. We hypothesize that any measurement error is likely independent of outdoor temperature, and that the observed associations between outdoor temperature and QTc underestimate the true associations. Finally, this study also consists of older men who are predominantly white, thus the observed findings may not be generalizable to women, younger individuals, or to other racial and ethnic groups.

In conclusion, we observed that increases in the daily mean temperature averaged over 3 to 4 weeks were associated with longer QTc, while increases in the daily variability of mean temperature averaged over 4 days and up to 4 weeks were associated with longer QTc, particularly in colder months and in individuals with diabetes and coronary heart disease. These novel findings may offer insight of an important underlying mechanism by which temperature affects cardiovascular events and mortality in an older population.

## Supporting Information

File S1 Figures S1a-S1e: Associations between QTc and 1 interquartile range increase of moving average 24-hr mean temperature measured from spatiotemporal predictive model by season (S1a) and by subgroups defined by diabetes (S1b), coronary heart disease (S1c), obesity (S1d), and age (S1e). Associations were estimated in linear mixed-effect regression models with random intercept for study participant and adjusted for years since baseline visit, age at baseline visit, race, body mass index, total cholesterol, mean arterial pressure, diabetes, QT prolonging medication, years of education, percent of census tract ≥25 years of age without high school diploma, percent of census tract that is non-white, alcohol consumption, smoking status, day of week, seasonality, 24-hour mean relative humidity, and 4-hour lag black carbon concentration; associations between moving average 24-hr mean temperature and QTc in each subgroup were estimated from interaction models.
(TIF)

Table S1 **Difference in QTc per unit increase in moving average 24-hr mean temperature and 24-hr standard deviation of temperature in fully and minimally adjusted models.**
(DOC)
