# Supplementary figures and images for: Associations between Changes in City and Address Specific Temperature and QT Interval - The VA Normative Aging Study
Source: PLoS One. 2014 Sep 19;9(9):e106258. doi: 10.1371/journal.pone.0106258 (PMC4169528; doi:10.1371/journal.pone.0106258)

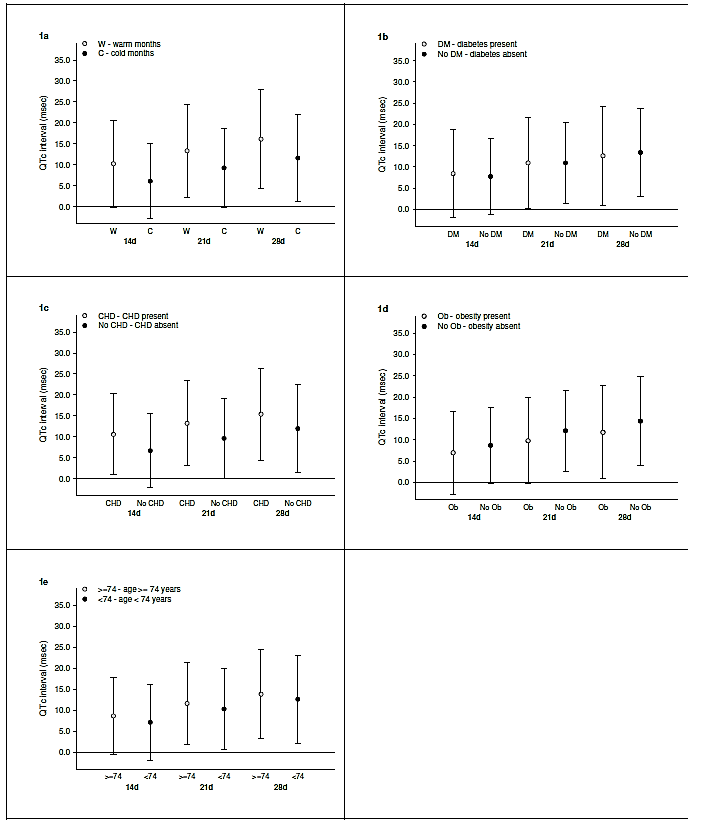

Supplement: File S1 — Figures S1a-S1e: Associations between QTc and 1 interquartile range increase of moving average 24-hr mean temperature measured from spatiotemporal predictive model by season (S1a) and by subgroups defined by diabetes (S1b), coronary heart disease (S1c), obesity (S1d), and age (S1e). Associations were estimated in linear mixed-effect regression models with random intercept for study participant and adjusted for years since baseline visit, age at baseline visit, race, body mass index, total cholesterol, mean arterial pressure, diabetes, QT prolonging medication, years of education, percent of census tract ≥25 years of age without high school diploma, percent of census tract that is non-white, alcohol consumption, smoking status, day of week, seasonality, 24-hour mean relative humidity, and 4-hour lag black carbon concentration; associations between moving average 24-hr mean temperature and QTc in each subgroup were estimated from interaction models. (TIF) [file pone.0106258.s001.tif]
